# Chemically-activatable alkyne-tagged probe for imaging microdomains in lipid bilayer membranes

**DOI:** 10.1038/srep41007

**Published:** 2017-01-24

**Authors:** Satoshi Yamaguchi, Taku Matsushita, Shin Izuta, Sumika Katada, Manami Ura, Taro Ikeda, Gosuke Hayashi, Yuta Suzuki, Koya Kobayashi, Kyoya Tokunaga, Yasuyuki Ozeki, Akimitsu Okamoto

**Affiliations:** 1 Research Center for Advanced Science and Technology (RCAST), The University of Tokyo, 4-6-1 Komaba, Meguro-ku, Tokyo, Japan; 2 Department of Chemistry and Biotechnology, Graduate School of Engineering, The University of Tokyo, 7-3-1 Hongo, Bunkyo-ku, Tokyo 113-8656, Japan; 3 Department of Electrical Engineering and Information Systems, Graduate School of Engineering, The University of Tokyo, 7-3-1 Hongo, Bunkyo-ku, Tokyo 113-8656, Japan

## Abstract

A chemically-activatable alkynyl steroid analogue probe has been synthesized for visualizing the lipid raft membrane domains by Raman microscopy. The Raman probe, in which ring A of its steroid backbone is replaced with an alkynyl group, was designed to enable activation of the alkyne signal through the Eschenmoser-Tanabe fragmentation reaction of the oxidized cholesterol precursor in lipid bilayer membranes. The alkynyl steroid analogue was observed to form liquid-ordered raft-like domains on a model giant-liposome system in a similar manner as cholesterol, and the large alkyne signal of the accumulated probe at 2120 cm^−1^ was mapped on the microdomains with a Raman microscope. The alkyne moiety of the probe was confirmed to be converted from the α,β-epoxy ketone group of its precursor by reaction with *p*-toluensulfonyl hydrazine under a mild condition. Through the reaction, the alkyne signal of the probe was activated on the lipid bilayer membrane of liposomes. Furthermore, the signal activation of the probe was also detected on living cells by stimulated Raman scattering microscopy. The ring-A-opened alkyne steroid analogue, thus, provides a first chemically-activatable Raman probe as a promising tool for potentially unravelling the intracellular formation and trafficking of cholesterol-rich microdomains.

Lateral segregation of subdomains in biological membranes, which is mainly characterized by enrichment of specific lipids such as cholesterol and sphingolipids is critical for exerting a range of cellular functions from membrane trafficking to signal transduction^1^,^2^. Such liquid-ordered membrane domains (“lipid rafts”) have been widely studied by visualization of the specific lipids both on the *in vitro* model membranes and cellular membranes^3^,^4^. However, although a number of fluorescence microscopic techniques with lipid-binding and lipid-analogous probes were employed, the details of the dynamics of domain formation and trafficking on the complex cellular membranes remained not to be well characterized as a result of a lack of appropriate probes^5^,^6^. Among the probes, fluorescent-labeled cholesterol analogs have been expected to visualize lipid rafts without disordering preexisting membrane structures^5^. In most cases, cholesterol is typically labeled with a fluorescent unit at the alkyl chain moiety, which is buried within the membrane^5^,^7^,^8^,^9^. Fluorescently labeled analogues, some of which have a charge, have been reported to be unsuitable in liquid-ordered membrane domains because of their steric and/or electrostatic repulsion between fluorescent labels in the membrane^10^. Therefore, images obtained from such fluorescent cholesterol might not accurately represent the original location and behavior of the cholesterol-rich raft domains. On the other hand, an intrinsic fluorescent sterol, dehydroergosterol (DHE), has the closest structure as cholesterol, but this analogue probe is difficult to be applied to real-time observation of living cells because its fluorescence requires short-wavelength UV irradiation and undergoes rapid photo-bleaching^11^,^12^.

Alkynes have recently attracted attention as a label to replace bulky charged fluorescent dyes. Alkynes are much smaller and they do not induce any intermolecular electrostatic interaction. In addition, carbon–carbon triple (C≡C) bonds in alkyne compounds can be used effectively for Raman imaging^13^,^14^,^15^. Raman microscopy can be used to image chemical moieties of intracellular molecules by recording the vibrational signatures of their characteristic bonds with Raman scattering of less-cytotoxic visible light. Moieties such as alkyne bonds and carbon–deuterium bonds, which rarely occur naturally, generate strong Raman signals in a region of the spectrum that is typically silent for cells (1800−2800 cm^−1^); that is, at frequencies at which most intracellular biomolecules show no Raman signals. Accordingly, alkyne-tagged small molecules can be visualized through the use of Raman microscopy without significant impact on the intrinsic properties of such compounds in cells^13^,^14^,^15^. Recently, a cholesterol analogue probe including C≡C bonds was reported to be utilized for visualizing cholesterol storage in living cells and *C. elegans*^16^. In this pioneering study, the static intracellular distribution of the cholesterol probe was observed by Raman imaging, comparable to those of conventional DHE by fluorescence imaging^11^,^16^.

Stimuli-responsive activatable probe has been reported to be a powerful tool for unravelling dynamic distribution changes of biomolecules^17^,^18^,^19^,^20^,^21^. A wide variety of activatable fluorescent probes that response to various stimuli such as light^17^, ions^18^,^19^, chemicals^20^ and enzymes^21^ have been actively studied. By using such probes, the labelled molecules which go through light-irradiated areas or specified environments such as low-pH, highly-reduced and enzyme-rich sites could be selectively visualized. In the latter cases, the selective visualization of the probes has yielded new insights into the microenvironment of the pathological regions, leading to development of new drug delivery and diagnosis systems^18^,^19^,^20^,^21^. Owing to the benefits of alkyne tag Raman imaging, an activatable alkyne probe would be potentially more promising tool for real-time observation in living systems. But, to our knowledge, no alkyne probes that are activated by external stimuli have been reported. Therefore, we tried to develop an activatable Raman probe for visualizing the dynamics of biomolecules. Herein, based on Eschenmoser–Tanabe fragmentation reaction, a chemically-activatable Raman probe for visualizing the cholesterol-rich microdomains is reported. Similar to cholesterol, this probe leads to the formation of liquid-ordered cholesterol-rich domains on the lipid bilayer membrane through preferential accumulation to the domains. In addition, this alkyne probe can be turned on from the Raman-silent state both on a model liposomal membrane and on living mammalian cells by chemical stimulus. Furthermore, in live-cell Raman imaging, activation of the present probe was observed to be accelerated under a mildly acidic condition due to low pH-enhanced conversion to the alkyne moiety^22^. These studies demonstrate the proof of principle that the present activatable probe may visualize the dynamics and trafficking of cholesterol-rich microdomains from the activation point of location particularly in a mildly acidic environment on living systems.

## Results

### Design and synthesis of alkyne-labeled steroid probe

**Figure 1:**
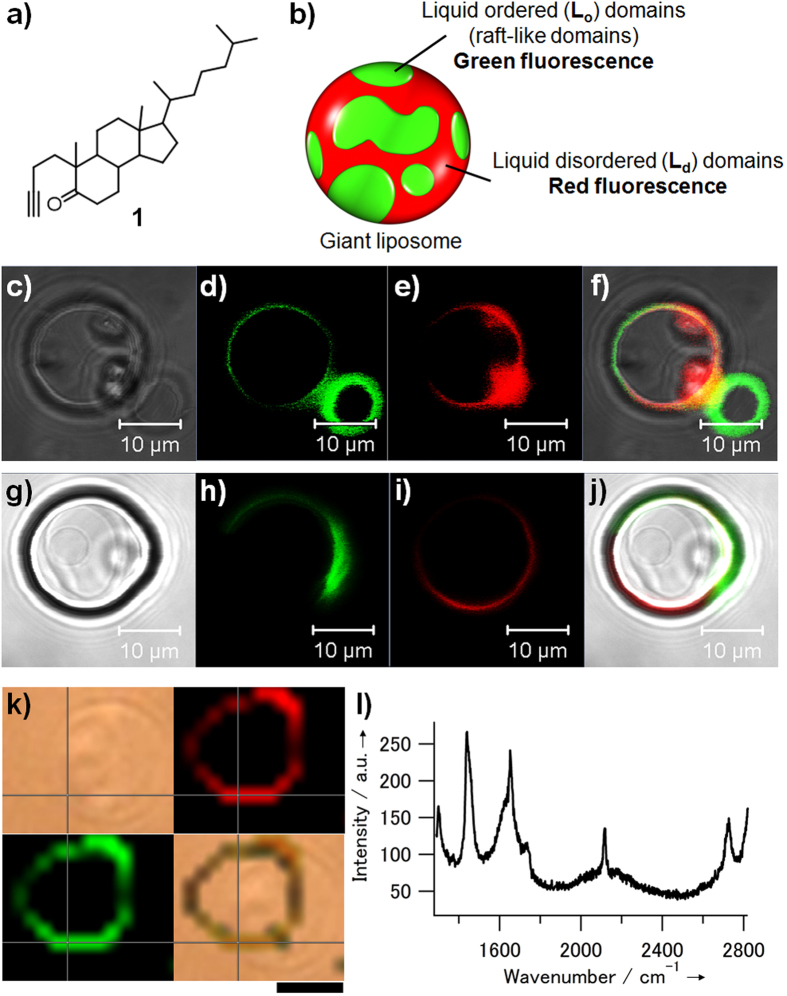
Domain formation properties and Raman microscopic imaging of the alkynyl steroid analogue probe on giant liposomes. (**a**) The chemical structure of the alkynyl steroid analogue probe **1**. (**b**) Illustration of a giant liposome (DOPC/DPPC/cholesterol or **1**, ratio 40:40:20) with liquid-ordered (L_o_) and liquid-disordered (L_d_) domains. These domains were stained with fluorescein-labeled Cholera toxin subunit B (green) and Rhodamine DHPE (red), respectively. (**c**–**j**) Fluorescence microscopic images of the giant liposomes consisting of (**c**–**f**) DOPC/DPPC/**1** and (**g**–**j**) DOPC/DPPC/cholesterol (40:40:20). (**c** and **g**) Differential interference contrast (DIC) images; (**d** and **h**) green fluorescence images; (**e** and **i**) red fluorescence images, and (**f** and **j**) their merged images. Scale bars: 10 μm. (**k**) Raman scattering images of the liposomes of DOPC/DPPC/**1** (40:40:20): (upper left) light-field images, (upper right) Raman scattering image at 1450 cm^−1^, (lower left) at 2120 cm^−1^, and (lower right) their merged images. Scale bars: 4 μm. (**l**) Raman spectrum obtained at the cross point on the liposome membrane in (**k**).

We first designed an alkyne-labeled steroid probe for enabling activation of the precursor in a lipid bilayer membrane. When steroids are inserted into a lipid bilayer membrane, ring D, tethering an alkyl chain, locates deep inside the membrane; accordingly, ring A lies toward the membrane surface. Thus, when considering conversion from the precursor that could be used after insertion in the membrane, ring A, which is located close to the membrane surface, would be a candidate structure because it would be an easily accessible reaction point on the steroid precursor molecule. Therefore, we designed alkynyl probe **1**, in which ring A of steroid is replaced with an alkynyl group (Fig. 1a). The probe **1** was synthesized efficiently through epoxidation and subsequent Eschenmoser–Tanabe fragmentation from 4-cholesten-3-one (Supplementary Scheme S1)^23^,^24^.

### Fluorescence microscopic analysis of microdomain formation on liposomes

Probe **1** was expected to form liquid-ordered domains on the membrane by preferentially accumulating on the domains. The distribution of **1** in the lipid domains of giant liposomes with a size of several micrometers was examined by using fluorescence microscopic imaging (Fig. 1b)^25^. Giant liposomes consisting of dioleoylphosphatidylcholine (DOPC), dipalmitoylphosphatidylcholine (DPPC), and **1** (40:40:20) were formed, and the liquid-ordered (raft-like) domains were stained with fluorescein-labeled Cholera toxin subunit B. The disordered membrane domain was stained fluorescently with Rhodamine DHPE. The giant liposome was also prepared by employing cholesterol instead of **1** as a positive control. For the DOPC/DPPC/**1** system, confocal microscopy confirmed that the two domains were separately stained with each fluorescently labeled probe (Fig. 1c–f). This result shows that probe **1** can form raft-like domains that are similar to those observed in cholesterol systems (Fig. 1g–j).

### Raman imaging and spectral analysis of alkyne-labeled steroid probe on lipid bilayer membranes

The alkynyl steroid probe **1** has a characteristic Raman signal that can be used for alkyne-tag Raman imaging. According to previous reports^13^,^14^,^15^, the distribution of alkynyl analogue **1** on the lipid bilayer membrane was visualized by mapping its characteristic Raman signal at 2120 cm^−1^ on the giant liposomes. Lipid membranes that were composed of DOPC (40%), DPPC (40%), and **1** (20%) were suspended in phosphate-buffered saline (PBS) solution with a small amount of biotin-modified poly(ethylene glycol)-lipid (0.20%). The biotinylated giant liposomes, which were immobilized on a streptavidin-coated quartz substrate on the dishes through biotin–streptavidin interaction^26^, were observed with a Raman microscope. The alkyne signal induced from **1** at 2120 cm^−1^ was clearly mapped to the liposome membrane and overlapped well with the signal corresponding to the C−C stretching vibration of lipid components at 1450 cm^−1^ (Fig. 1k). Furthermore, in the Raman images, there were a few domains where both the C≡C and C−C signals were relatively large. The Raman spectra obtained at a selected point on the domain exhibited large Raman signals arising from both C≡C and C−C stretching vibrations (Fig. 1l).

Steroid probe **1** exhibited different behavior in living cells to that observed in the liposome experiments. Cholesterol-rich domains in living cells are known to be internalized from plasma membranes into the endoplasmic reticulum in the endocytic pathways^1^. The distribution of **1** in living HeLa cells was investigated by employing stimulated Raman scattering (SRS) after cell uptake of **1** by using methyl β-cyclodextrin (MβCD)^12^. SRS images at 2090, 2115, 2140, and 2930 cm^−1^ of HeLa cells treated with **1** were obtained. In the image at 2115 cm^−1^, granular signals were observed from the cytosolic region, especially around the nucleus (Supplementary Figure S3).

### Conversion to alkyne-tagged steroid probe in aqueous-based media

**Figure 2:**
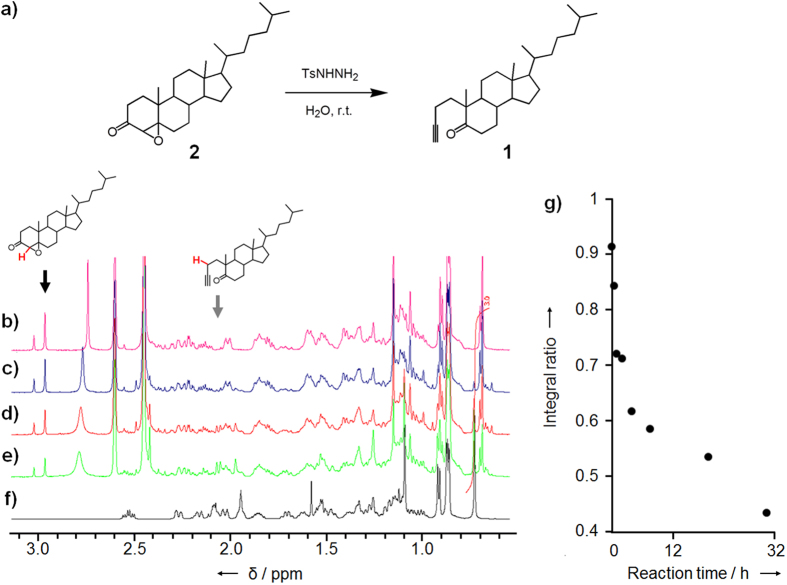
Chemical activation of the steroid analogue probe with the alkyne moiety in aqueous solutions. (**a**) Alkyne formation of steroid analogue probe **1**. (**b**–**f**) ^1^H NMR measurement of production of alkynyl probe **1** from precursor **2**. The ^1^H NMR spectra of **2** were obtained after incubation with TsNHNH_2_ in D_2_O/DMSO for (**b**) 0, (**c**) 2.0, (**d**) 7.5, and (**e**) 31 h. (**f**) The ^1^H NMR spectrum of **1** was obtained as positive control. The black arrow indicates the chemical shifts of the proton of **2** that is marked in red in the chemical structure shown above the spectra (2.97 and 3.02 ppm of enantiomers). The gray arrow indicates the chemical shift of the red-colored proton indicated in **1** (2.06 ppm). (**g**) The integral ratio of the chemical shifts of the red-colored proton indicated in **2** (indicated by the black arrow) was plotted as a function of the reaction time.

Next, the alkyne moiety of **1** was examined to be generated under physiological conditions. As described above, it was designed to be produced through Eschenmoser–Tanabe fragmentation, which was used in the second step for the preparation of **1**. In the fragmentation reaction, an aliphatic ring containing an α,β-epoxy ketone moiety is opened by addition of *p*-toluenesulfonyl hydrazine (TsNHNH_2_), resulting in the production of an alkyne group. In our case, 4,5-epoxycholestan-3-one (**2**), was converted in ethanol into ring-A-opened alkyne **1** (Supplementary Scheme S1)^24^. Developing this approach further, we confirmed that this reaction also proceeds very well in aqueous-based media at room temperature (Fig. 2a). Alkyne-precursor **2** was mixed with TsNHNH_2_ in a mixture of deuterated water and deuterated dimethyl sulfoxide (40% conc.) and the solution was incubated for various periods of time at room temperature. ^1^H NMR spectroscopic analysis revealed that as the specific chemical shifts of **1** appeared, shifts corresponding to **2** simultaneously decreased (Fig. 2b–f). The peak intensity of the epoxy ketone (2.97 and 3.02 ppm of diastereomers) decreased to 60% within 5 h (Fig. 2g). The IR spectrum revealed a signal at 2118 cm^−1^ corresponding to the formation of the alkyne moiety after incubation of the present aqueous-based system for 4 h (Supplementary Figure S4).

Alkyne formation occurs efficiently even on a lipid bilayer membrane. We prepared liposomes including **2** and investigated the alkyne formation reaction on the liposomes and subsequent labeling with an azide-substituted fluorescein through copper-catalyzed click chemistry (Huisgen cycloaddition)^27^. Only after treatment with TsNHNH_2_, emission from the tethering fluorescein was clearly detected on the pellet of liposomes after centrifugation (Supplementary Figure S5a–c). The fluorescence was also observed on the lipid membrane of the liposomes under a microscope (Supplementary Figure S5d–f).

### Chemical activation of the Raman signal of alkyne-tagged steroid probe

**Figure 3:**
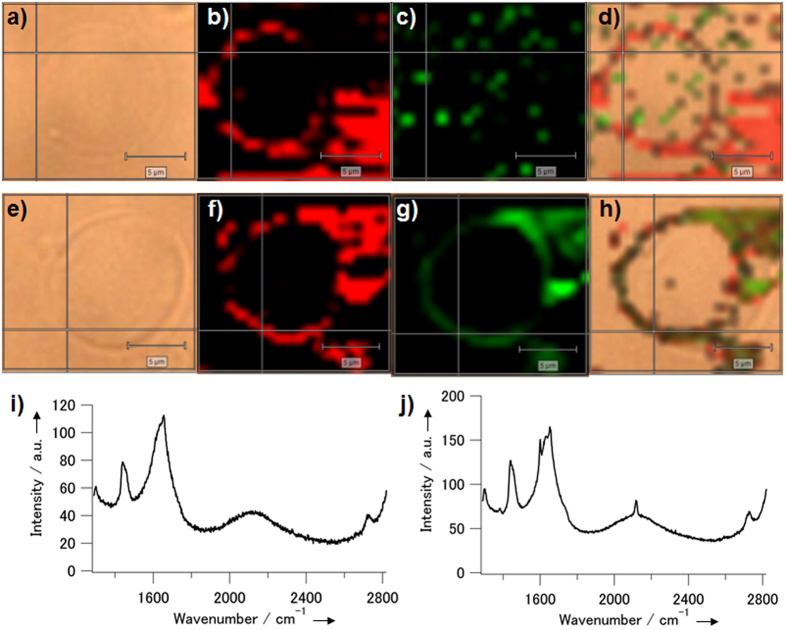
Raman microscopic imaging of the liposomes including the chemically-activatable alkynyl steroid analogue probe before and after activation. Microscopic images of the liposomes including **1** were obtained (**a**–**d**) before and (**e**–**h**) after incubation with TsNHNH_2_. The light field images (**a** and **e**), the Raman scattering images at 1400 cm^−1^ (**b** and **f**) and at 2120 cm^−1^ (**c** and **g**), and their merged images (**d** and **h**) are shown. Scale bars: 5 μm. (**i** and **j**) Raman spectra obtained at the cross point on the liposome membrane in (**a**) and (**e**), respectively.

The Raman signals of **1** formed by chemical conversion of **2** on membrane were also observed by using the giant liposomes as described above. The giant liposomes consisting of DOPC (40%), DPPC (40%), and **2** (20%) were immobilized onto the substrate of the dishes, and were observed with a Raman microscope before and after treatment with TsNHNH_2_ for 40 min. After treatment with TsNHNH_2_, a sharp signal appeared at 2120 cm^−1^ in the Raman spectra of the whole image including the liposome, which were obtained by multicomponent analysis (Supplementary Figure S6). In contrast, before TsNHNH_2_ treatment, no signal was observed at the same wavenumber in the major components separated by multicomponent analysis. Thus, the Raman signal contributed by the chemical conversion of **2** into **1** was confirmed to be detected at 2120 cm^−1^. Furthermore, to visualize activation of the alkyne signal on the lipid bilayer membranes alone, the Raman signals at 2120 cm^−1^ were mapped before and after treatment with TsNHNH_2_. Before treatment with TsNHNH_2_, only noise was observed in the image recorded at the alkyne stretching frequency, whereas the C−C signal at 1400 cm^−1^ was correctly mapped to the liposome membrane (Fig. 3a–d). After treatment with TsNHNH_2_, the alkyne signal was clearly mapped to the liposome membrane and overlapped perfectly with the C−C signal (Fig. 3e–h). In the Raman spectrum obtained at a selected point on the membrane, peaks due to both C≡C and C−C stretching vibrations were confirmed, in contrast to the spectrum obtained before treatment with TsNHNH_2_ (cf. Fig. 3i and j).

**Figure 4:**
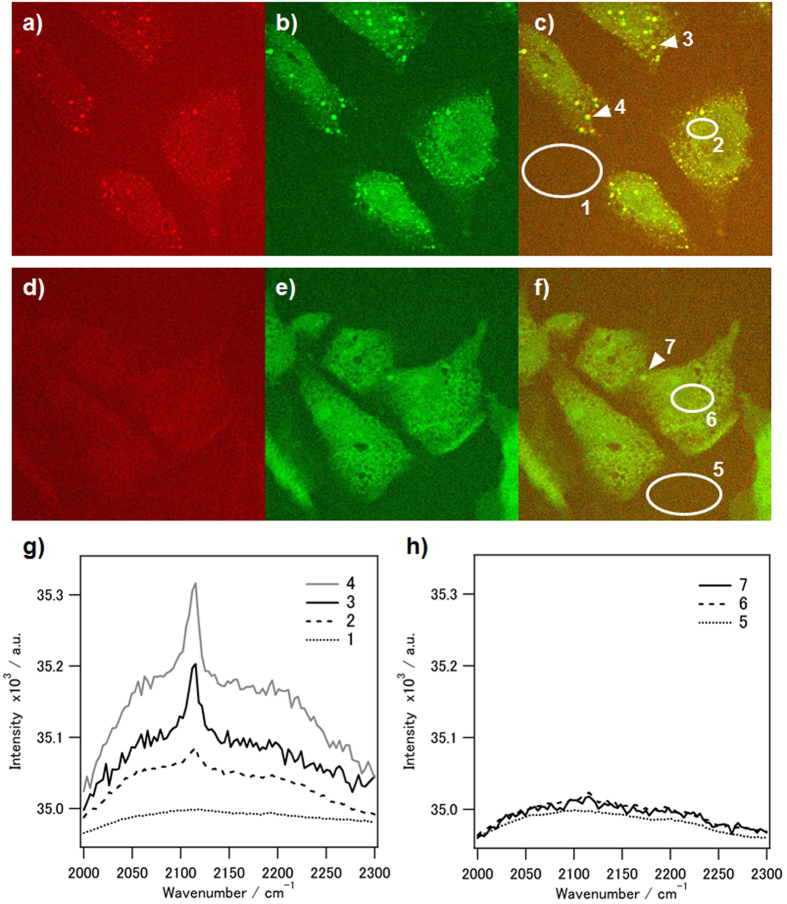
Stimulated Raman scattering (SRS) images of HeLa cells treated with chemically-activatable alkynyl steroid analogue probe and activated with TsNHNH_2_ under (**a**–**c**) mildly acidic and (**d**–**f**) neutral conditions. The SRS images at (**a** and **d**) 2115 and (**b** and **e**) 2930 cm^−1^, and (**c** and **f**) the merged images at 2115 and 2930 cm^−1^ were obtained. The white circles and arrows indicate the areas and spots where the distinctive SRS spectra were obtained by further analysis. (**g**) The SRS spectra obtained from the circular areas with the number of 1 (black dotted line) and 2 (black broken line) and the spots 3 (black line) and 4 (grey line) in fig. 4c, respectively. (**h**) The SRS spectra obtained from the circular areas with the number of 5 (black dotted line) and 6 (black broken line) and the spot 7 (black line) in fig. 4f, respectively.

Finally, alkyne formation was also observed on living cells by SRS microscopy. The precursor **2** was incorporated into the cellular membranes of HeLa cells in almost the same way as alkyne steroid probe **1**. After treatment with TsNHNH_2_ for 30 min, SRS images at the wavenumbers of 2115 cm^−1^ and 2930 cm^−1^ were obtained for visualizing the distributions of the activated alkyne probe. In the image at 2930 cm^−1^, the whole cell region can be simply visualized by mapping the signal of C-H bonds in lipids and proteins^28^. On the image, the granular signals on which the SRS spectra have the clear peak at 2115 cm^−1^ were observed from the intracellular region when the treatment with TsNHNH_2_ was performed under a mildly acidic condition (Fig. 4a–c and g). By contrast, no clear signal was observed from the cell treated with TsNHNH_2_ at a neutral pH in the image at 2115 cm^−1^ (Fig. 4d–f and h). Thus, the activation of the C≡C stretching vibration signal was confirmed in live-cell imaging, and under the present experimental condition, the activated signals were selectively observed from the cells in a mildly acidic medium. Here, the influences of the probe and TsNHNH_2_ on cell viability were investigated with a commercially-available viability assay kit, Cell counting kit-8 (CCK-8) assay. We confirmed that incorporation of the precursor steroid analogue **2** into cellular membranes with MβCD had no influence on the viability, and incubation with TsNHNH_2_ for 30 min briefly decreased the viability to approximately 80% (Supplementary Figure S8).

## Discussion

In this study, a ring-A-opened alkyne steroid analogue probe has been synthesized as a first chemically-activatable Raman probe. From the pioneer studies of Sodeoka’s group^13^,^14^, alkyne-tag Raman imaging is a powerful approach for visualizing small biomolecules in living systems for a long period without perturbing their intrinsic properties. If the alkyne group tagged on the molecules could be generated by biorthogonal stimuli under physiological conditions, the alkyne-tagged probe would be more valuable as an activatable Raman probe for stimuli-responsive selective visualization. However, as evidenced by the fact that compounds containing C≡C bonds exist very rarely in cells, it is difficult to obtain a C≡C bond from its precursor under physiological conditions through the use of external stimuli. In looking for a suitable reaction that could be used to generate the alkyne moiety, we focused on reactions that proceed well under mild conditions. We focused in particular on Eschenmoser–Tanabe fragmentation^23^,^24^, where an alkyne group is converted from a stable epoxy ketone moiety by addition of TsNHNH_2_. In the previous reports, this reaction proceeded in polar solvents such as alcohols at slightly high temperatures. Furthermore, TsNHNH_2_ is water soluble, and to our knowledge, the severe cytotoxicity of TsNHNH_2_ has not been reported. Accordingly, it seemed to be promising that the fragmentation reaction was employed as the alkyne generation reaction in living systems. Thus, we decided to employ the oxidized cholesterol **2** containing an α,β-epoxy ketone moiety, which can be converted to the alkyne steroid probe **1** through the fragmentation reaction.

The alkyne steroid probe **1** was demonstrated to form microdomains on lipid bilayer membranes. Formation of the liquid-ordered microdomains which enrich cholesterol and saturated phospholipid has been reported to occur spontaneously in giant liposomes containing cholesterol, saturated phospholipid, and unsaturated phospholipid^29^. The microdomains are widely considered to be analogous to lipid raft in cell membranes. In the model membranes, the formation of the raft-like domains is highly dependent on cholesterol composition because the driving force of the lateral segregation is the difference in the affinity of cholesterol for the two phospholipids: the molecular flatness of the rigid sterol ring favors interaction with straighter and stiffer hydrocarbon chains of saturated lipids and disfavors interaction with the more bulky unsaturated ones^2^. In this study, the liquid-ordered and disordered domains were separately stained by membrane domain-specific fluorescent probes in giant liposomes containing DOPC (40%), DPPC (40%), and **1** (20%) (Fig. 1). When the content rate of **1** was decreased to 13%, the domain separation was not observed (Supplementary Figure S1). This result strongly suggested that the ring-A-opened steroid structure of **1** contributed to the domain formation in the similar manner as cholesterol. Furthermore, in the Raman image of the liposome, there are the domains where both the C≡C and C−C signals were relatively large (Fig. 1k). This is probably because the large signals were derived from the accumulated **1** and highly-packed DPPC in the liquid-ordered domains. From these results, the alkyne steroid probe **1** was suggested to behave as the analogue of cholesterol in the formation of microdomains. Similar to cholesterol and **1**, the precursor steroid analogue **2** was also confirmed to lead to the microdomain formation (Supplementary Figure S2). From the results, the present activatable probe was suggested not to drastically alter their distribution to the microdomains before and after activation.

Conversion of the precursor steroid analogue **2** into the alkyne analogue **1** was demonstrated in aqueous-based media and on liposomes. The precursor **2** has low solubility in water because of its hydrophobicity. Accordingly, the conversion reaction with TsNHNH_2_ was monitored by ^1^H-NMR in a mixture of deuterated water and deuterated DMSO (40%). As a result, the reaction was confirmed to proceed at room temperature (Supplementary Figure S4), although it seemed to stop at about 40% conversion (Fig. 2). This is probably because the precursor **2** was not completely dissolved into the reaction mixture solution. Actually, in our further trial using a different water-soluble derivative, most of the α,β-epoxy ketone moiety was converted to the alkyne product in an aqueous solution within 1 h, and in particular, the conversion was almost finished within 15 min in a mildly acidic aqueous solution^22^. In Fig. 2g, a part of precursor **2** also seemed to be rapidly converted to **1** at the beginning of the reaction within a few hours because the dissolved fraction of **2** could quickly react with TsNHNH_2_. Thus, alkyne formation through the ring-opening reaction of epoxy ketone proceeded in the aqueous-based media efficiently in a short time as long as the precursor moiety was homogenously diffused. The same conversion was investigated on the lipid bilayer membranes for showing the possibility of its application to cellular membrane imaging. Alkyne formation on the membrane was preliminarily detected by fluorescence labelling through click reaction with an azide-modified dye. Selective fluorescent staining of the liposomal membrane including the precursor **2** was observed after treatment with TsNHNH_2_ (Supplementary Figure S5). This result indirectly suggested the progress of the alkyne formation reaction on the membranes. Therefore, to directly indicate the alkyne formation, the liposomal membrane including **2** was observed by Raman microscopy before and after TsNHNH_2_ treatment (Fig. 3). The Raman signals derived from the alkyne moiety at 2120 cm^−1^ was clearly mapped to the membrane in the image obtained after TsNHNH_2_ treatment (Fig. 3g), whereas only noise was observed in the image at 2120 cm^−1^ before TsNHNH_2_ treatment (Fig. 3c). This result directly shows that precursor **2** reacted on the liposome membrane in the presence of TsNHNH_2_ to form alkyne **1**.

The activatable steroid probe was applied to live-cell Raman imaging of cholesterol-rich domains. According to previous reports^12^, the steroid analogue probes were introduced onto the plasma membranes through MβCD-mediated replacement of the inherent cholesterol with the probes. In the preliminary experiment, we introduced alkyne steroid probe **1** into HeLa cells and observed the intracellular distribution of the alkyne signal by spontaneous Raman microscopy. However, the alkyne signal was only weakly detected from the cell even by increasing the cumulated number of scanning. To obtain the image with both higher sensitivity and higher speed, the cells were observed with the SRS imaging system^28^. As a result, alkyne-derived granular signals were clearly observed from the intracellular cytosolic region in the image at 2115 cm^−1^ (Supplementary Figure S3). Cholesterol-rich raft domain is well known to internalize from the plasma membrane to the intracellular domain via the endocytosis pathway^1^. After steady-state distribution in living cells within 30 min, the signal of cholesterol analogue probes was often observed from plasma membrane (PM), endocytic recycling compartment (ERC) and lipid droplets^30^: the signals on PMs appeared as a diffuse, hazy one; those on ERCs and lipid droplets were bright granular spots and larger punctate structures, respectively. In our case, only the signals of the alkyne steroid probe on granular or punctate organelle were clearly detected probably due to its higher concentration than that on PMs (Supplementary Figure S2). In a previous report^16^, the alkyne signals of the similar cholesterol probe were also observed as granular spots in the cytoplasm by SRS microscopy, and it was reported to show accumulation of the probes in lipid droplets. Similarly, in the case of precursor probe **2**, the granular signals with the peak at 2115 cm^−1^ were observed in the cytoplasm after TsNHNH_2_ treatment under a mildly acidic condition (Fig. 4a–c). This result strongly suggests that some of precursor **2** was converted to alkyne steroid probe **1** through the alkyne formation reaction on cellular membranes, followed with endosomal internalization to lipid droplets. Thus, chemical activation from the Raman-silent state of the ring-A-closed precursor was possible even on living cells. It is noteworthy that the alkyne signal was not only observed from the granular spots but also from the cytosol area by optimizing the conditions of SRS imaging such as the laser intensity, although the intensity of the cytosol signal was relatively low compared to the granular signals (Fig. 4c and g, area 2). Furthermore, the alkyne signal in the cytosol disappeared after incubation in a culture medium for 2 h, suggesting that the cholersterol-rich domains on intracellular traffic were localized to granular lipid droplets (Supplementary Figure S7). These results suggest that the distribution change of the present steroid probe can be visualized after chemical activation. By contrast, such activation of alkyne signals was not observed after TsNHNH_2_ treatment at pH7.4 (Fig. 4d–f). In our other work, the rate of alkyne formation was shown to be almost five times slower under neutral conditions than under mildly acidic ones^22^. Accordingly, it is assumed that the conversion rate at pH7.4 was too low to give the visible alkyne signals in the SRS images under the present experimental condition. This result strongly suggests that the present activatable steroid probe can be applied to selective visualization of the cholesterol-rich domains which are treated with TsNHNH_2_ in a mildly acidic environment in living systems. It is well-known that a decrease in pH can occur locally in specific intracellular organelles such as endosomes and lysosomes or in certain pathological regions, cancer tissues and inflammatory sites^31^,^32^. Therefore, low-pH-activatable imaging probes have been studied in the fields of bioanalytical and biomedicinal chemistry^18^,^33^, and actually, were applied to selectively visualize low-pH lung tumors *in vivo* to assist in surgical planning and operations^18^. The present low-pH-activatable steroid probe is also potentially applicable to live-cell or *in vivo* Raman imaging of the trafficking and distribution of cholesterol-rich domains under/from acidic environments in living systems. In addition, it was shown from the results of the cell viability assay that the present probes and the activation procedures do not have large impact on the cell viability (Supplementary Figure S6).

The present probe is the first activatable Raman probe for the cholesterol-rich domains in cellular membranes, and potentially applicable to visualization of their dynamic localization through chemical pulse activation. Chemical activation generally depends on the environmental factors such as temperature and pH, and the local conditions such as the reactant concentration, permeability and diffusion. Accordingly, the membrane domains which exist in a specified environment in living systems may be selectively visualized, and the domains only in a limited area are potentially visualized by localizing the reactant hydrazine through its modification with a targeting moiety or a photo-removable protection group. The present alkyne steroid probe thus will be a platform tool to take a big step toward producing some activatable imaging methods for understanding the intracellular distribution pathways and the environment-specific dynamics of the cholesterol-rich raft-like domains.

## Methods

### Synthesis of probes

Precursor steroid probe **2** was synthesized through epoxidation of 4-cholesten-3-one (from Wako Chemicals, Osaka, Japan), and alkynyl steroid probe **1** was synthesized from **2** through Eschenmoser–Tanabe fragmentation reaction^24^. The details of the synthec procedures were shown in Supplementary Information.

### Domain formation on giant liposomes

Fluorescently-stained raft-exhibiting giant liposomes were prepared essentially according to a previous report^25^. The films of the lipid mixture (DOPC/DPPC/cholesterol or **1**/_D_-(+)-glucose = 2:2:1:15) with 0.2% Rhodamin-DHPE and 1% GM_1_ was prepared in glass tubes, and hydrated with deionized water, followed with incubation at 37 °C for one hour to form giant vesicles. The liposome suspension was combined with a solution of fluorescein-labeled CtxB-488 for staining the cholesterol-rich raft-like domain. The fluorescent images of liposomes were acquired using a confocal fluorescent microscope (LSM 510 META confocal microscopy, Carl Zeiss, Jena, Germany).

### Evaluation of the chemical activation with ^1^H-NMR spectroscopy

^1^H-NMR spectra of the precursor **2** were measured after incubation with TsNHNH_2_ in aqueous-based media for various periods. To a solution of **2** in DMSO-*d*_6_ (0.4 mL) was added a solution of TsNHNH_2_ (20 mM) in D_2_O (0.6 mL). After mechanically shaking for several hours at room temperature (Micro Mixer E-36, from TaiTec, Tokyo, Japan), the solution was extracted with chloroform-*d* (0.6 mL). The ^1^H-NMR spectra were measured with Avance 600 (600 MHz, from Bruker, Germany).

### Raman imaging of probe-introduced giant liposomes

Raft-exhibiting giant liposomes consisting of the lipid mixture (DOPC/DPPC/**1** or **2**/D-(+)-glucose = 2:2:1:15) with 0.2% biotin-modified poly(ethylene glycol)-lipid were prepared as described above. The biotinylated giant liposomes were immobilized on a streptavidin-coated quartz substrate on the dishes through biotin–streptavidin interaction^26^. In the case of **2**, a solution of TsNHNH_2_ in MilliQ (20 mM) was added onto the dishes for chemical conversion of **2** to **1** on the liposome. After treatment with TsNHNH_2_ for 40 min, the TsNHNH_2_ solution was removed, and the surface was rinsed with MilliQ water. The bright field images and Raman images were acquired with a confocal unit Renishaw inVia Raman Microscope (Renishaw plc, UK). The acquired images were analyzed and processed for multicomponent analysis with the software WiRE 4.1 (Renishaw plc, UK).

### SRS imaging of probe-introduced living cells

Either the synthesized alkynyl steroid probe **1** or the precursor probe **2** was introduced into HeLa cells. The cultured cells were treated with the mixed solution of **1** (40 mM) or **2** (10 mM) with MβCD (100 or 150 mM) in PBS (100 μL) for 2 min. After treatment, the culture dish surfaces were rinsed with PBS twice. In the case of **2**, the cells were treated with DMEM including 20 mM TsNHNH_2_ for 30 min to activate the alkyne signal of the probe. The pHs of the TsNHNH_2_ solutions were adjusted to pH6.4 and 7.4. The images of the cells were obtained with the SRS imaging system, which was previously reported^28^.

## Additional Information

**How to cite this article**: Yamaguchi, S. *et al*. Chemically-activatable alkyne-tagged probe for imaging microdomains in lipid bilayer membranes. *Sci. Rep.* **7**, 41007; doi: 10.1038/srep41007 (2017).

**Publisher's note:** Springer Nature remains neutral with regard to jurisdictional claims in published maps and institutional affiliations.

## Supplementary Material

Supplementary Information

## References

[b1] Simons K. & Ikonen E. Functional rafts in cell membranes. Nature 387, 569–572 (1997). 9177342 10.1038/42408

[b2] Lingwood D. & Simons K. Lipid rafts as a membrane-organizing principle. Science 327, 46–50 (2010). 20044567 10.1126/science.1174621

[b3] Cicuta P., Keller S. L. & Veatch S. L. Diffusion of liquid domains in lipid bilayer membranes. J. Phys. Chem. B. 111, 3328–3331 (2007). 17388499 10.1021/jp0702088

[b4] Ishitsuka R., Sato S. B. & Kobayashi T. Imaging lipid rafts. J. Biochem. 137, 249–254 (2005). 15809325 10.1093/jb/mvi041

[b5] Sengupta P., Hammond A. & Baird B. Structural determinants for partitioning of lipids and proteins between coexisting fluid phases in giant plasma membrane vesicles. Biochim. Biophys. Acta 1778, 20–32 (2008). 17936718 10.1016/j.bbamem.2007.08.028 PMC2679899

[b6] Makino A. . Visualization of the heterogeneous membrane distribution of sphingomyelin associated with cytokinesis, cell polarity, and sphingolipidosis. FASE. J. 29, 477–493 (2015). 10.1096/fj.13-247585 25389132

[b7] Baumgart T. . Fluorescence probe partitioning between L_o_/L_d_ phases in lipid membranes. Biochim. Biophys. Acta 1768, 2182–2194 (2007). 17588529 10.1016/j.bbamem.2007.05.012 PMC2702987

[b8] Scheidt H. A. . The potential of fluorescent and spin-labeled steroid analogs to mimic natural cholesterol. J. Biol. Chem. 278, 45563–45569 (2003). 12947110 10.1074/jbc.M303567200

[b9] Li Z., Mintzer E. & Bittman R. First synthesis of free cholesterol-BODIPY conjugates. J. Org. Chem. 71, 1718–1721 (2006). 16468832 10.1021/jo052029x

[b10] Ramirez D. M. C., Ogilvie W. W. & Johnston L. J. NBD-cholesterol probes to track cholesterol distribution in model membranes. Biochim. Biophys. Acta 1798, 558–568 (2010). 20026044 10.1016/j.bbamem.2009.12.005

[b11] Mukherjee S. . Cholesterol distribution in living cells: fluorescence imaging using dehydroergosterol as a fluorescent cholesterol analog. Biophys. J. 75, 1915–1925 (1998). 9746532 10.1016/S0006-3495(98)77632-5 PMC1299862

[b12] Gimpl G. & Gehrig-Burger K. Cholesterol reporter molecules. Biosci. Rep. 27, 335–358 (2007). 17668316 10.1007/s10540-007-9060-1

[b13] Yamakoshi H. . Imaging of EdU, an alkyne-tagged cell proliferation probe, by Raman microscopy. J. Am. Chem. Soc. 133, 6102–6105 (2011). 21443184 10.1021/ja108404p

[b14] Yamakoshi H. . Alkyne-tag raman imaging for visualization of mobile small molecules in live cells. J. Am. Chem. Soc. 134, 20681–20689 (2012). 23198907 10.1021/ja308529n

[b15] Wei L. . Live-cell imaging of alkyne-tagged small biomolecules by stimulated Raman scattering. Nat. Methods 11, 410–414 (2014). 24584195 10.1038/nmeth.2878 PMC4040164

[b16] Lee H. J. . Assessing cholesterol storage in live cells and *C. elegans* by stimulated Raman scattering imaging of phenyl-diyne cholesterol. Sci. Rep. 5, 7930 (2015). 25608867 10.1038/srep07930 PMC4302291

[b17] Ando R., Mizuno H. & Miyawaki A. Regulated fast nucleocytoplasmic shuttling observed by reversible protein highlighting. Science 306, 1370–1373 (2004). 15550670 10.1126/science.1102506

[b18] Urano Y. . Selective molecular imaging of viable cancer cells with pH-activatable fluorescence probes. Nat. Med 15, 104–109 (2009). 19029979 10.1038/nm.1854 PMC2790281

[b19] Hirata T. . Protein-coupled fluorescent probe to visualize potassium ion transition on cellular membranes, Anal. Chem. 88, 2693–2700 (2016). 26894407 10.1021/acs.analchem.5b03970

[b20] Izumi S. . A simple and effective strategy to increase the sensitivity of fluorescence probes in living cells, J. Am. Chem. Soc. 131, 10189–10200 (2009). 19572714 10.1021/ja902511p

[b21] Asanuma D. . Sensitive β-galactosidase-targeting fluorescence probe for visualizing small peritoneal metastatic tumours *in vivo*, Nat. Commun. 6, 6463 (2015). 25765713 10.1038/ncomms7463 PMC4382686

[b22] Yamaguchi S. . Chemically activatable alkyne tag for low pH-enhanced molecular labeling on living cells. *Bioconj. Chem.* 27, 1976–1980 (2016). 10.1021/acs.bioconjchem.6b00399 27526276

[b23] Felix D. . α,β-Epoxyketon → alkinon-fragmentierung I: synthese von exalton und rac - muscon aus cyclododecanon über synthetische methoden, 3. Mitteilung Helv. Chim. Acta 54, 2896–2912 (1971).

[b24] Corey E. J. & Sachdev H. S. 2,4-Dinitrobenzenesulfonylhydrazine, a useful reagent for the Eschenmoser. α, β cleavage of α, β-epoxy ketones. Conformational control of halolactonization J. Org. Chem. 40, 579–581 (1975).

[b25] Hamada T. . Dynamic processes in endocytic transformation of a raft-exhibiting giant liposome. Phys. Chem. Lett. B 111, 10853–10857 (2007). 10.1021/jp075412+ 17718558

[b26] Lohse B., Bolinger P. & Stamou D. Encapsulation efficiency measured on single small unilamellar vesicles. J. Am. Chem. Soc. 130, 14372–14373 (2008). 18842043 10.1021/ja805030w

[b27] Rostovstev V. V. . A stepwise Huisgen cycloaddition process: copper (I)-catalyzed regioselective ligation of azides and terminal alkynes. Angew. Chem. Int. Ed. 41, 2596–2599 (2002). 10.1002/1521-3773(20020715)41:14<2596::AID-ANIE2596>3.0.CO;2-4 12203546

[b28] Ozeki Y. . High-speed molecular spectral imaging of tissue with stimulated Raman scattering. Nat. Photon. 6, 845–851 (2012).

[b29] Veatch S. L. & Keller S. L. Organization in lipid membranes containing cholesterol. Phys. Rev. Lett. 89, 268101 (2002). 12484857 10.1103/PhysRevLett.89.268101

[b30] Mondal M. . Sterols are mainly in the cytoplasmic leaflet of the plasma membrane and the endocytic recycling compartment in CHO cells. Mol. Biol. Cell. 20, 581–588 (2009). 19019985 10.1091/mbc.E08-07-0785 PMC2626560

[b31] Rotin D., Robinson B. & Tannock I. F. Influence of hypoxia and an acidic environment on the metabolism and viability of cultured cells: potential implications for cell death in tumors. Cancer Res. 46, 2821–2826 (1986). 3698008

[b32] Tannock I. F. & Rotin D. Acid pH in tumors and its potential for therapeutic exploitation. Cancer Res. 49, 4373–4384 (1989). 2545340

[b33] Tóth E. . Water-soluble gadofullerenes: toward high-relaxivity, pH-responsive MRI contrast agents. J. Am. Chem. Soc. 127, 799–805 (2005). 15643906 10.1021/ja044688h

