# Supplementary material for: Chemically-activatable alkyne-tagged probe for imaging microdomains in lipid bilayer membranes
Source: Sci Rep. 2017 Jan 24;7:41007. doi: 10.1038/srep41007 (PMC5259774; doi:10.1038/srep41007)
Supplement: Supplementary Information [file srep41007-s1.pdf]

Supplementary Information

Chemically-activatable alkyne-tagged probe for imaging microdomains in lipid bilayer membranes

Satoshi Yamaguchi[1], Taku Matsushita[2], Shin Izuta[2], Sumika Katada[2], Manami Ura[2], Taro Ikeda[2], Gosuke Hayashi[2], Yuta Suzuki[3], Koya Kobayashi[3], Kyoya Tokunaga[3], Yasuyuki Ozeki[3] and Akimitsu Okamoto[1,2*]

[1]Research Center for Advanced Science and Technology (RCAST), The University of Tokyo, 4-6-1 Komaba, Meguro-ku, Tokyo, Japan

[2]Department of Chemistry and Biotechnology, [3]Department of Electrical Engineering and Information Systems, Graduate School of Engineering, The University of Tokyo, 7-3-1, Hongo, Bunkyo-ku, Tokyo 113-8656, Japan

E-mail: okamoto@chembio.t.u-tokyo.ac.jp

1. Synthesis of alkynyl steroid probe 1

Alkynyl steroid probe **1** was synthesized in two steps from a commercially available starting compound **3** (4-cholesten-3-one, from Wako Chemicals, Osaka, Japan),) (Scheme S1).

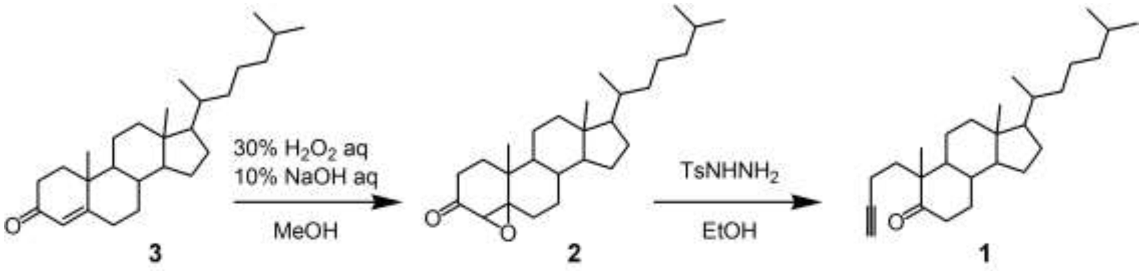

Scheme S1. Synthesis of the precursor steroid probe **2** and the alkynyl steroid probe **1**.

General procedures and materials. All reagents were commercially available and used as supplied without further purification. Column chromatography was performed on a silica gel provided by Kanto Chemical Co. Inc. (60N spherical, 40−50 μm). TLC was performed on Merck aluminum-backed plates coated with silica gel $60F_{254}$. Spots were visualized by heating with anisaldehyde. NMR chemical shifts are reported in ppm downfield of tetramethylsilane using residual solvent as an internal reference. NMR spectra were recorded by using Avance 600 (600 MHz, from Bruker, Germany). FT-IR spectra were recorded with a FT/IR-6100 type A (JEOL, Japan). ESI mass spectra were recorded with a micrOTOF II (Bruker, Germany). MALDI-TOF spectra were recorded with microflex (Bruker, Germany).

Synthesis of 2. To a solution of **3** (2.50 g, 6.51 mmol) in methanol (300 ml) were added 30% hydrogen peroxide solution (18 mL, 176 mmol) and 10% sodium hydroxide solution (6 mL, 167 mmol). After stirring for 49 h at 0 ºC, the solution was poured into water (180 mL), extracted with dichloromethane (150 mL) three times and further extracted with dichloromethane (50 mL) three times, and evaporated. After evaporation, the crude product was purified by column chromatography on silica gel (hexane/ethyl acetate = 20 / 1) to give **2** (1.54 g, 59 %) as a white powder. TLC R_f: 0.70 (hexane/ethyl acetate = 4, green spot after heating with anisaldehyde).^{1}H-NMR (600 MHz, $CDCl_3$, TMS): δ 2.99 (1H ,s), 2.30 (1H, dd), 2.21 (1H, m), 2.13 (1H, m), 2.00 (1H, d), 1.85 (3H), 1.58 (3H, s), 1.40−1.32 (5H, dd), 1.25 (1H, m), 1.20−1.00 (m, 12H), 0.90 (3H, t), 0.85 (d, 6H), 0.68 (d, 3H). ESI-MS (*m/z*): 399.46 $[M-1]^-$.

Synthesis of 1. To a solution of **2** (422 mg, 1.05 mmol) in ethanol (30 mL) was slowly added a solid of *p*-toluenesulfonyl hydrazine (264 mg, 1.41 mmol). After stirring for 2 h at room temperature, the solvent was removed under vacuum. The crude product was purified by column chromatography on silica gel (hexane/ethyl acetate = 20 / 1) to give **1** (230 mg, 57 %) as a colorless oil. TLC R_f: 0.70 (hexane/ethyl acetate = 4, purple spot after heating with anisaldehyde). ^{1}H-NMR (600 MHz, $CDCl_3$, TMS): δ 2.51 (1H, m), 2.26 (1H, m), 2.16 (1H, dd), 2.09 (2H, m), 2.03 (1H, m), 1.94 (2H, m), 1.85 (1H, m), 1.70 (1H, q), 1.63 (1H, q), 1.58 (1H, s), 1.49 (4H, m), 1.35 (3H, m), 1.26 (3H, m), 1.22−0.95 (10H, m), 0.92 (3H, d), 0.86 (6H, dd), 0.74 (3H, s), ESI-MS (*m/z*): 385.31$[M+1]^+$.

2. Preparation and fluorescent imaging of fluorescently-stained raft-exhibiting giant liposomes

Fluorescently-stained raft-exhibiting giant liposomes were prepared essentially according to a previous report.[S1] The lipid mixture (dioleylphosphatidylcholine (DOPC)/dipalmitoylphosphatidylcholine(DPPC)/cholesterol, **1** or **2**/D-(+)-glucose = 2:2:1:15) with 0.2% rhodamine B-dihexadecanoylphosphatidylamine (rhodamin-DHPE) (λ_{ex} = 560 nm, λ_{em} = 580 nm) and 1% GM_1 in 1:2 (*v/v*) chloroform/methanol was dried under vacuum for 24 ~ 39 h to form lipid films in glass tubes. The films were hydrated with 1 mL of deionized water and incubated in water bath at 37 °C for one hour to form giant vesicles. The final total concentration of lipid mixture (DOPC/DPPC/cholesterol or **1**) was 0.1 mM. The liposome suspension was combined with the same volume of 25 µg/mL fluorescein-labeled Cholera toxin subunit B (CtxB-488) (λ_{ex} = 495 nm, λ_{em} = 519 nm) in deionized water for staining the cholesterol-rich raft-like domain. The liposomes spontaneously adsorbed onto the glass bottom of the dishes, and then, fluorescent images were acquired using a confocal fluorescent microscope (LSM 510 META confocal microscopy, Carl Zeiss, Jena, Germany).

Similarly, the giant liposomes that were composed of DOPC (43.5%), DPPC (43.5%), and **1** (13%) were prepared and stained as described above. Figure S1 shows the confocal fluorescent images of the giant liposome (DOPC/DPPC/**1**, ratio 43.5:43.5:13). Different from the giant liposomes containing **1** at a higher rate (DOPC/DPPC/**1**, ratio 40:40:20) (Fig. 1c-f), the domain separation was not observed in this system.

In addition, the giant liposomes were prepared by mixing DOPC (40%), DPPC (40%), and **2** (20%). Figure S2 shows their confocal images. Similar to cholesterol and **1**, the two microdomains on the liposomes were observed to be separately stained by rhodamin-DHPE and CtxB-488.

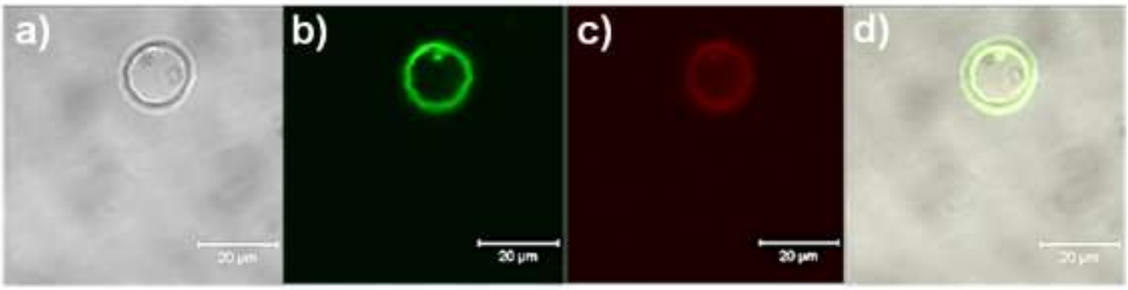

Figure S1. Fluorescent microscopic images of the giant liposome consisting of DOPC/DPPC/**1** (43.5:43.5:13). (a) Differential interference contrast (DIC) image; (b) green fluorescent image of fluorescein-labeled CtxB-488; (c) red fluorescent image of rhodamin-DHPE, and (d) their merged image.

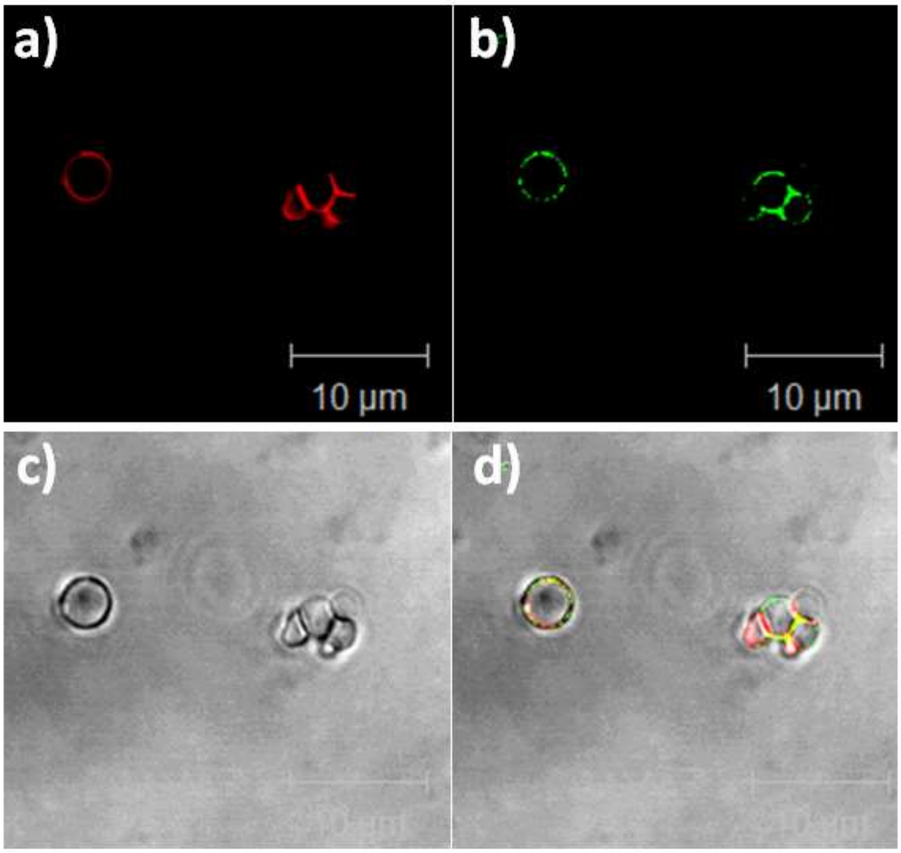

Figure S2. Fluorescent microscopic images of the giant liposome consisting of DOPC/DPPC/**2** (40:40:20). (a) Differential interference contrast (DIC) image; (b) green fluorescent image of fluorescein-labeled CtxB-488; (c) red fluorescent image of rhodamin-DHPE, and (d) their merged image.

3. Synthesis of biotin-modified PEG-lipid for biotinylation of liposomal surfaces

DOPE-PEG-biotin **9** was synthesized in three steps from a commercially available starting compound **4** (dioleylphosphatidylethanolamine (DOPE), from Wako Chemicals, Osaka, Japan) and **5** (Boc-PEG-NHS, from NOF Co., Ltd, Tokyo, Japan) (Scheme S2).

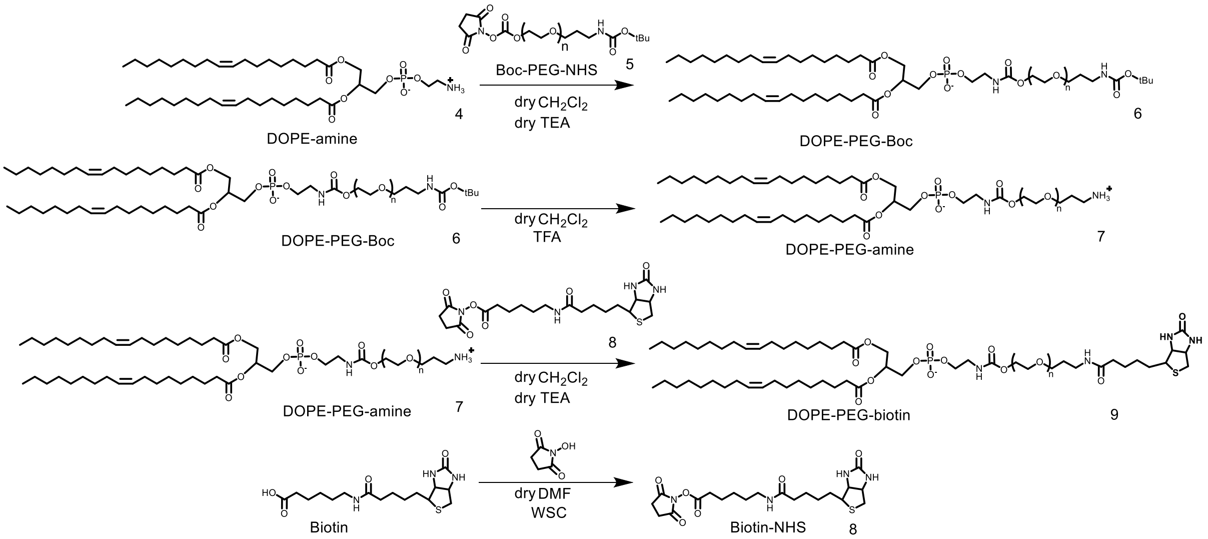

Scheme S2. Synthesis of DOPE-PEG-biotin **9**

Synthesis of 6. The lipid **4** (24.2 mg, 1.4 eq, 33μmol) and a polyethyleneglycol linker **5** was dried up and displaced with argon gas. To the solid were slowly added dry dichloromethane (5.0 mL) and triethylamine (50 μL), stirred at room temperature for 18.5 h and poured into cooled diethylether (0 °C, 100 mL). The suspension was centrifuged at 10 krpm for 10 min at 0 °C. The supernatant was removed by decantation, and then the residual white solid was air-dried overnight. DOPE-PEG-Boc (**6**) (125 mg 92%) was yielded as a white solid after drying *in vacuo*. TLC R_f: 0.45 (chloroform / methanol = 6:1, a white spot after heating with anisaldehyde). ^{1}H-NMR (600 MHz, $CDCl_3$, TMS): δ 5.55 (1H ,s), 5.36 (4H, s), 5.23 (1H, s), 5.04 (1H, m), 4.39 (1H, d), 4.21 (3H, d), 4.01 (2H, m), 3.41 (2H, m), 3.25 (2H, t), 3.12 (2H, m), 2.33(4H, q), 2.04 (8H, m), 1.76 (2H, q), 1.60 (4H, m), 1.54~1.18 (49H, m), 0.90 (6H, t). MALDI-TOF-MS (*m/z*): 5696.72 $[M-1]^-$.

Synthesis of 7. Compound **6** (120 mg, 21 μmol) was dried up and displaced with argon gas. To the solid were slowly added dry dichloromethane (4.0 mL) and triefluoroacetic acid (100 μL), stirred at room temperature for overnight and evaporated, yielding DOPE-PEG-amine (**7,** 170 mg, quant.) as a clear oil after drying *in vacuo*. TLC R_f: 0.50

(chloroform/methanol = 6:1, a white spot after heating with anisaldehyde). ^{1}H-NMR (600 MHz, $CDCl_3$, TMS): δ 5.37 (4H, s), 5.24 (1H, s), 5.17 (1H, m), 4.37 (1H, d), 4.25 (3H, m), 4.15 (2H, m), 3.46 (4H, m), 3.17(2H, m), 2.31(4H, q), 2.05 (8H, m), 1.94 (2H, q), 1.60 (4H, m), 1.53−1.16 (40H, m), 0.86 (6H, t). MALDI-TOF-MS (*m/z*): 5650.66 (M−1).

Synthesis of 9. Compound **7** (130 mg, 23 μmol) was dried up and displaced with argon gas. To the solid was added dry dichloromethane (4.0 mL) and triethylamine (100 μL). Biotin-NHS (**8**) (24.4 mg, 56 μmol) was dried up and displaced with argon gas. To the solid was added dry dichloromethane (1.0 mL). These two solutions were mixed and stirred for 62 h, and evaporated, yielding DOPE-PEG-biotin (**9,** 76 mg, 58%) as a white solid after drying *in vacuo*. TLC R_f: 0.60 (chloroform/methanol = 6:1, a white spot after heating with anisaldehyde). ^{1}H-NMR (600 MHz, $CDCl_3$, TMS): δ 5.36 (4H, t), 5.21 (1H, t), 4.36 (1H, m), 4.23 (1H, m), 4.18 (1H, dd), 3.98 (3H, m), 3.38 (4H, m), 3.18 (1H, q), 2.92 (1H, d), 2.75 (1H, d), 2.50 (4H, q), 2.16 (2H, m), 2.03 (8H, m), 1.46 (4H, m), 1.40−1.14 (40H, m), 0.89 (6H, t). MALDI-TOF-MS (*m/z*): 5873.51$[M-1]^{-}$.

4. Preparation and Raman imaging of raft-exhibiting giant liposomes

Raft-exhibiting giant liposomes were prepared essentially accoring to a previous report.[S1] The lipid mixture (DOPC/DPPC/**1**/$_{D}$-(+)-glucose = 2:2:1:15) with 0.2% **9** in 1:2 (*v/v*) chloroform/methanol was dried under vacuum for 12 h to form lipid films in glass tubes. The giant vesicles were prepared from the lipid films as described above. The present raft-exhibiting giant liposomes were employed for Raman imaging.

A streptavidin-coated surface were prepared for immobilizing biotinylated liposomes according to a previous report.[S2] First, the bottoms of 35-mm quartz-based dishes (FPI, Kyoto, Japan) were coated with bovine serum albumin (BSA) by treating with the 0.1% BSA solution in Dulbecco’s PBS, which included biotin-BSA (from Life Technologies Japan, Tokyo, Japan) at the rate of 10%. After incubation for 15 min at room temperature, the dish surface was rinsed with PBS twice, followed by drying. Then, 0.1 mL of 25 μM streptavidin in PBS was placed onto the BSA-coated dishes. After incubation for 10 min at room temperature, the dish surface was rinsed with 1 mL of PBS twice. The present streptavidin-coated dishes were used for immobilizing biotinylated liposomes in Raman imaging.

The raft-exhibiting giant liposomes with the biotin moiety on their surfaces were immobilized onto the streptavidin-coated dishes through the biotin−streptavidin

interaction. The 1 mL of the liposome suspension was placed on the streptabidin-coated dish. After incubation for 6 h, the suspension was removed, and the dish surface was rinsed with 400 µL of MilliQ water. The bright-field images and Raman images were acquired with a confocal unit Renishaw inVia Raman Microscope (Renishaw plc, UK). The acquired images were analyzed and processed for multicomponent analysis with the software WiRE 4.1 (Renishaw plc, UK).

5. Stimulated Raman scattering imaging of the living cells treated with alkynyl steroid analogue

Human cervical cancer (HeLa) cells were cultured in Dulbecco's modified Eagle medium (DMEM) containing 10% heat-inactivated fatal bovine serum (FBS) at 37 ºC under a humidified atmosphere with 5 % CO_2. For experimental use, cells were cultured on quartz-base dishes. After removal of DMEM, the dish surfaces were rinsed with 1 mL of PBS twice. After washing, the mixed solution of alkynyl steroid analogue **1** (40 mM) and methyl β-cyclodextrin (MβCD) (100 mM) in PBS (100 µL) was added onto the dish surface. After incubation for 2 min, the surfaces were rinsed with 1 mL of PBS twice, and then 1.0 mL of DMEM (without phenol red) was poured into the dish. Then, the cells were observed with the stimulated Raman scattering (SRS) imaging system, which was previously reported.[S3]

Figure S3 shows the SRS images of **1**-treated HeLa cells at the wavenumbers of 2090, 2115, 2140 and 2930 cm^{-1}. Here, the image at 2930 cm^{-1} was obtained for visualizing the whole cells with the signal from C-H in lipids and proteins. In the image at 2115 cm^{-1}, the granular signals were clearly observed from the cytosolic region, while no signal was observed at 2090 and 2140 cm^{-1}. From these results, the observed signal at 2115 cm^{-1} was derived from the alkyne moiety of **1**.

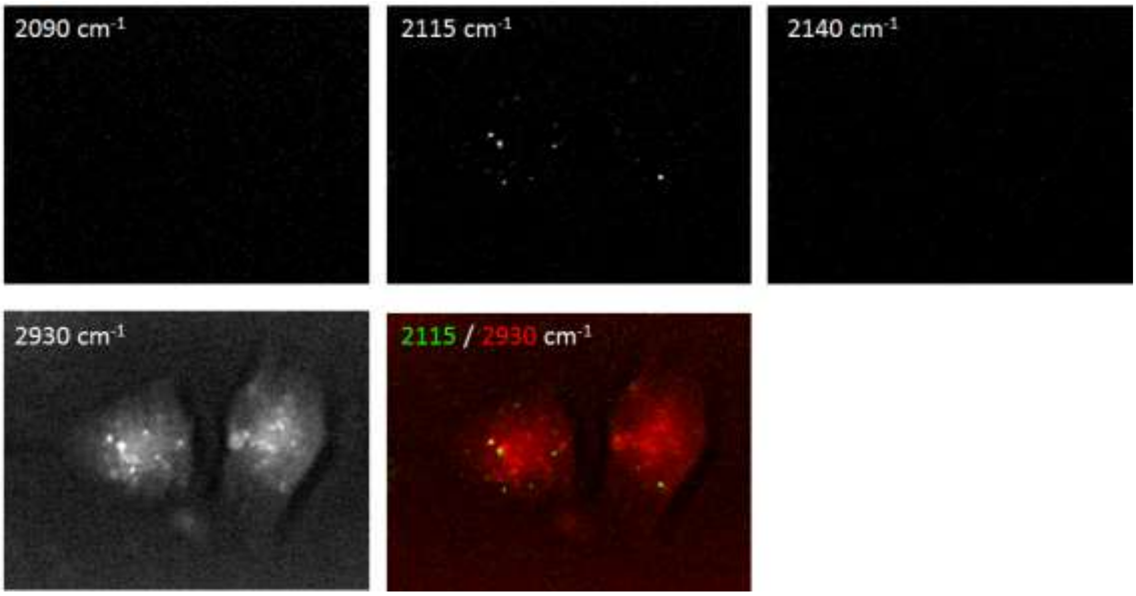

Figure S3. Stimulated Raman scattering (SRS) images of HeLa cells after treatment with alkynyl steroid analogue **1**. The SRS signal images at 2090 cm^{-1} (top left), 2115 cm^{-1} (top center), 2140 cm^{-1} (top right) and 2930 cm^{-1} (bottom left), and the merged image at 2115 and 2930 cm^{-1} (bottom center).

6. Evaluation of the chemical activation with ^{1}H-NMR spectroscopy

^{1}H-NMR spectra of the alkynyl steroid precursor **2** were measured after incubation with *p*-toluenesulfonyl hydrazine ($TsNHNH_2$) in aqueous-based media. To a solution of **2** (concentrated) in DMSO-d_6 (0.4 mL) in 2.0 mL tube was added a solution of $TsNHNH_2$ (20 mM) in D_2O (0.6 mL). After mechanically shaking for several hours at room temperature (Micro Mixer E-36, from TaiTec, Tokyo, Japan), the solution was extracted with chloroform-*d* (0.6 mL). This solution was put into a glass NMR tube.

7. Evaluation of the chemical activation with FT-IR spectroscopy

To a solution of **2** (concentrated) in DMSO-d_6 (0.4 mL) in 2.0 mL tube was added a solution of $TsNHNH_2$ (20 mM) in D_2O (0.6 mL). After mechanically shaking for 4 h as described above, the product in the solution was precipitated by adding 1 mL of MilliQ water, followed by centrifugation. The supernatant was removed by decantation. After washing with 2 mL of MilliQ water, vacuum drying the precipitation yielded a colorless oil. FT-IR spectra were obtained by potassium bromide disk method.

Figure S4 shows the FT-IR spectra obtained by potassium bromide disk method. Comparing these spectra, the peak derived from alkyne group was confirmed to appear at 2118 cm^{-1} (Fig. S4, indicated by the black arrow).

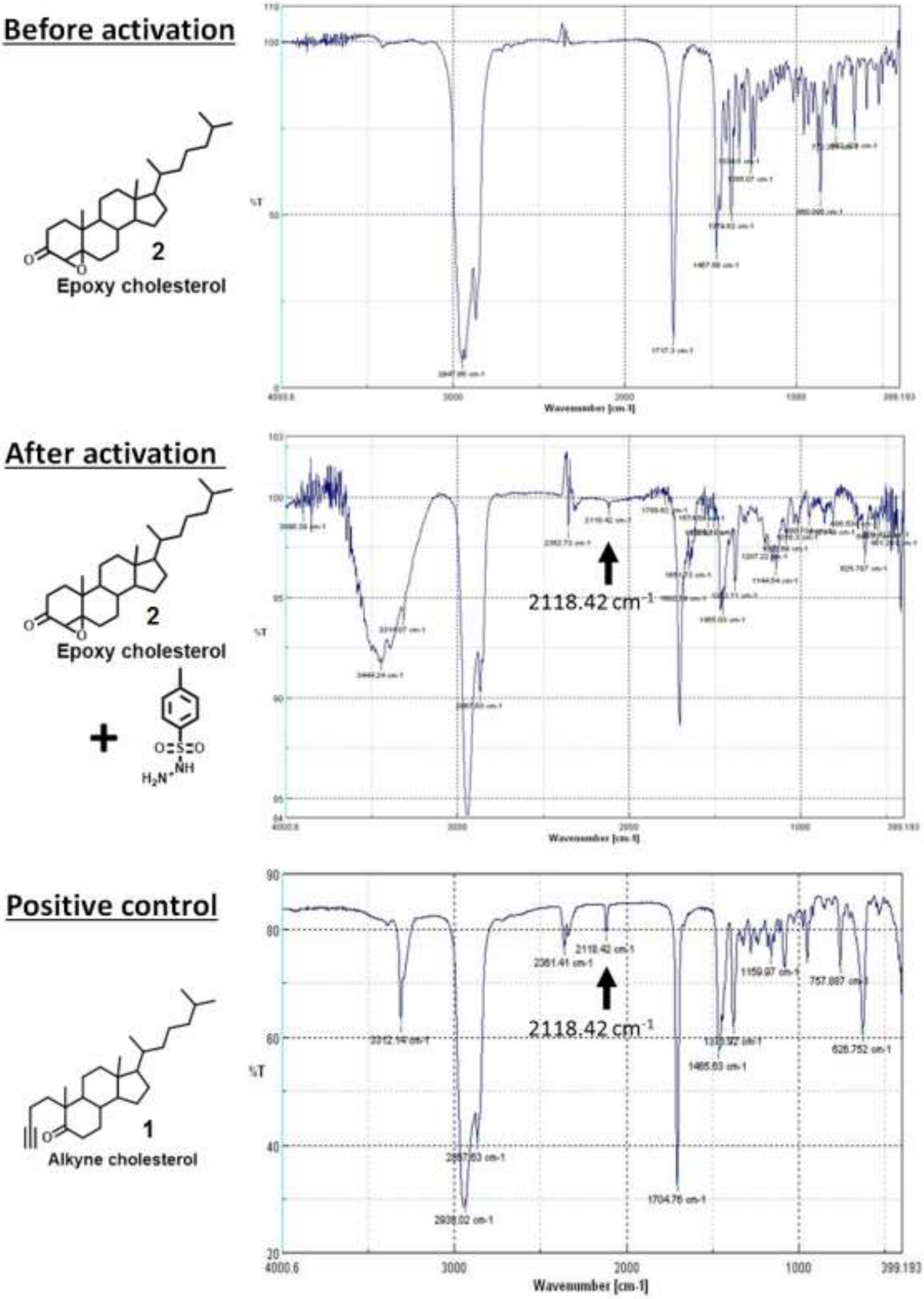

Figure S4. FT-IR spectra of **2** obtained before and after incubation with *p*-toluenesulfonyl hydrazine in water solvent for 4 h (top and middle) and that of **1** obtained as the positive control (bottom).

8. **Preparation of giant liposomes including the alkynyl steroid precursor and chemical activation.**

Giant liposomes were prepared according to a previous report.[S4] To a solution of lethicin (6.1 mg, 8 μmol), cardiolipin (0.6 mg) and **2** (3.2 mg, 8 μmol) in chloroform (4 mL) was slowly added MilliQ water (7 mL) and then, chloroform was removed from the bilayer solution by evaporation at 100 hPa for 1 h to prepare a giant liposome suspension. To the liposome suspension (2 mL) was added 5-azidefluorescein (3.4 mM) in MilliQ water (6.7 μL), copper(II) sulfate (50 mM) in MilliQ water (50 μL), sodium ascorbate (50 mM) in MilliQ water (100 μL) and $TsNHNH_2$ (20 mM) in MilliQ (500 μL). After stirring for 6 h, a 0.1 mL-portion of the liposome suspension was placed onto the bottoms of 35-mm plastic dishes. Fluorescent images (λ_{ex} = 488 nm) of the liposomes were acquired using a confocal fluorescence microscope (LSM 510 META, from Zeiss, Germany). The liposomes in the residual suspension was precipitated by centrifugation and then, the supernatant was removed with a pipette. After washing with MilliQ water three times, the photographs of the liposomal precipitation were acquired.

As a result, only the case with treatment of both $TsNHNH_2$ and the dye, the yellow color derived from the dye was clearly observed on the pellet of liposomes (Fig. S5a–c). Similarly, only on the sample, the fluorescence of the dye was microsopically observed on the lipid membrane of the liposomes (Fig. S5d-f). From these results, it was suggested that $TsNHNH_2$-induced alkyne formation on the lipid membrane led to successive modification of the dye through copper-catalyzed Hüsgen cyclization.

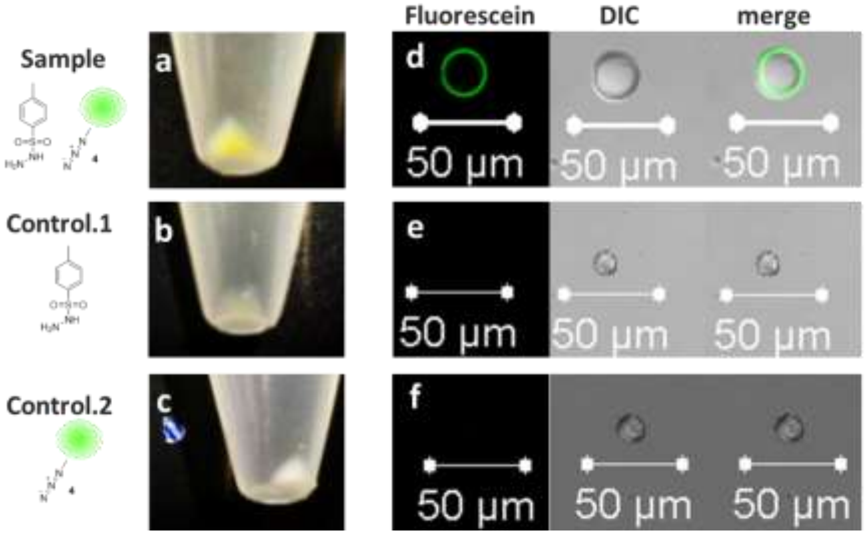

Figure S5. Photographs and fluorescence microscopic images of the liposomes after treatment of $TsNHNH_2$ and 5-azidefluorescein with a copper-catalized click reaction reagents. (a−c) The photographs of the pellets of liposomes and (d−f) the fluorescence

microscopic images (λ_{ex} = 488 nm) of liposomes. (a,d) Sample was reacted both with $TsNHNH_2$ and 5-azidefluorescein. (b,e) Control 1 reacted only with $TsNHNH_2$ and, (c,f) Control 2 reacted only with 5-azidefluorescein. Scale bars: 50 µm.

9. Raman imaging of chemical activation of the alkynyl steroid probe on giant liposomes

The biotinylated giant liposomes consisiting of DOPC (40%), DPPC (40%) and **2** (20%) were prepared and immobilized onto the substrate of the dishes as described above. For chemical conversion of **2** to **1** on the liposomes, the 1 mL solution of $TsNHNH_2$ in MilliQ (20 mM) was added onto the dishes. After treatment with $TsNHNH_2$ for 40 min, the $TsNHNH_2$ solution was removed, and the surface was rinsed with MilliQ water. The bright-field images and Raman images were acquired and analyzed as described above.

Figure S6 shows the Raman spectra of the top six components obtained by multicomponent analysis of the whole area of a liposome image. In the first and second components of the image observed after $TsNHNH_2$ treatment, the peak apeared at the wavenumber of 2120 cm^{-1}. On the other hand, no peak was detected at the same wavenumber in the major six components before $TsNHNH_2$ treatment.

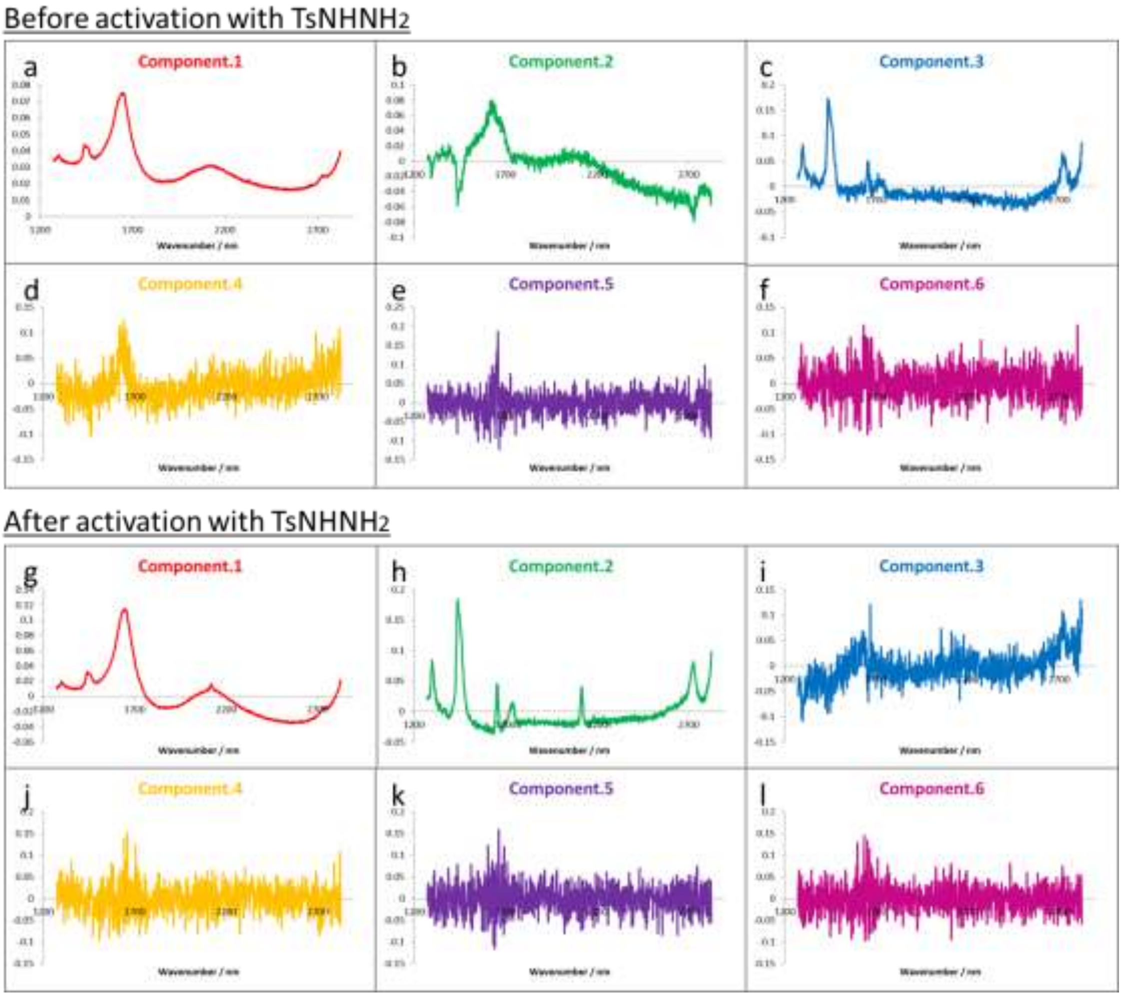

Figure S6. Raman spectra of the major components obtained by multicomponent analysis of giant liposomes including **2**. (a−f) Before $TsNHNH_2$ treatment and (g−l) after $TsNHNH_2$ treatment. The panels of the top six components were arranged on the basis of relative rankings of the components in multicomponent analysis. (a, g) Component 1, (b, h) component 2, (c, i) component 3, (d, j) component 4, (e, k) component 5 and (f, l) component 6.

10. Stimulated Raman scattering imaging of the treated cells after chemical activation

HeLa cells were cultured on quartz-base dishes as described above. After washing, the mixed solution of precursor oxidized cholesterol **2** (10 mM) and MβCD (150 mM) in PBS (20% DMF) was added onto the dish surface. After incubation for 2 min, the surfaces were rinsed, and then DMEM (without phenol red) including 20 mM

$TsNHNH_2$, in which pHs were adjusted to pH6.4 or pH7.4, was poured into the dish, followed with incubation for 30 min. Then, the cells were observed before and after incubation in the culture medium (DMEM with 10% FBS) for 2h at 37 ºC under 5% CO_2 with the stimulated Raman scattering (SRS) imaging system as described above.

Figure S7 shows the SRS images of the cells after incubation for 2h. From the image of the cells treated under a mildly acidic condition (Fig. S7a-c), the granular alkyne signals were observed, but the homogenous alkyne signal was not clearly observed from the cytosol, different from the image before incubation (Fig. 4a).

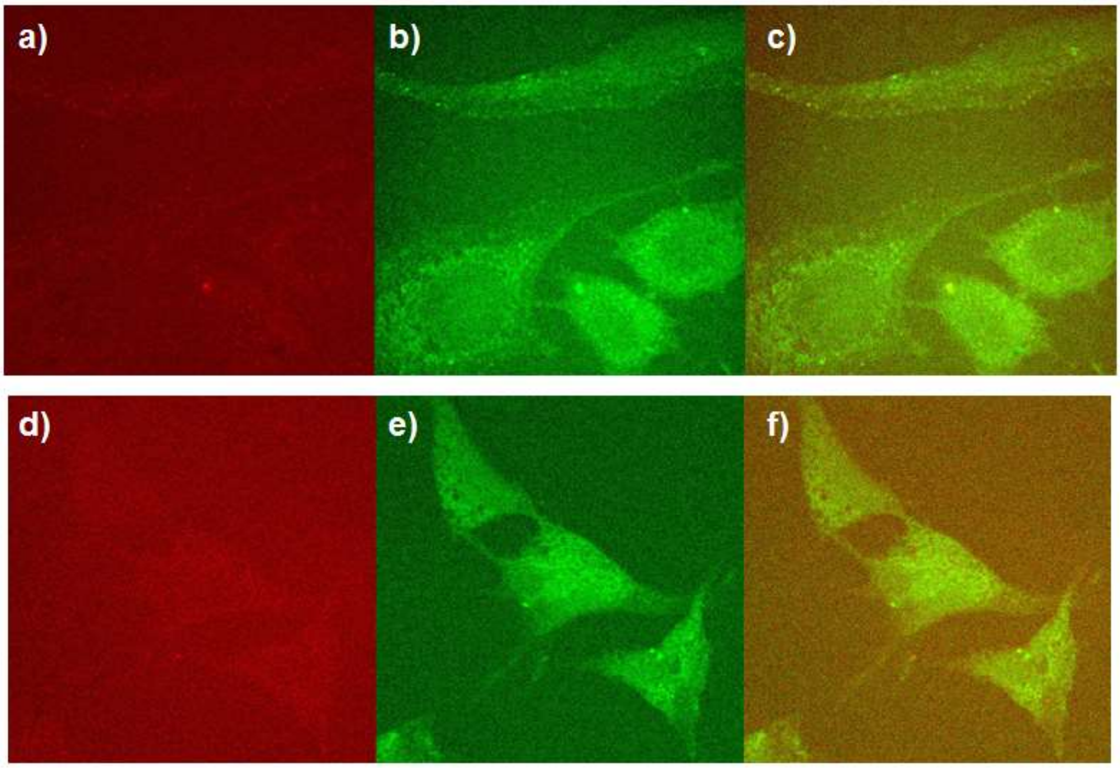

Figure S7. Stimulated Raman scattering (SRS) images of HeLa cells after incubation for 2h. Before incubation, the cells were treated with chemically-activatable alkynyl steroid analogue probe and activated with $TsNHNH_2$ under (a-c) mildly acidic and (d-f) neutral conditions. The SRS signal images at (a and d) 2115 and (b and e) 2930 cm-1, and the merged images at 2115 and 2930 cm-1 were obtained.

11. Cell viability test

The viablities of the treated HeLa cells were evaluated by a commercially-available tetrazolium metabolism-based assay kit, Cell Counting Kit-8 (CCK-8) assay (Dojindo Molecular Technologies, Inc., Tokyo). According to the protocol of the kit, HeLa cells were cultured in culture medium (DMEM including 10% FBS) in 96-well culture dishes for 1 day, and then, were treated with a precursur **2** solution for 2 min and with a $TsNHNH_2$ solution for 30 min as described in the previous section (section 10). After culture in the culture medium for 48 hours, the CCK-8 reagent was added into each well. After incubation for 30 min, the absorbance of each well at 450 nm was measured by a plate-reader. To evaluate the influence of the reagents, the viabilities were normalized with the adsorbance of the control cell sample which was treated with PBS for 2 min and with culture medium.

Figure S8 shows that the flash treatment of the mixture of precursor **2** and MβCD had no influence on the cell viability. In addition, the treatment of the $TsNHNH_2$ solution was shown to briefly decrease the viability to almost 80%.

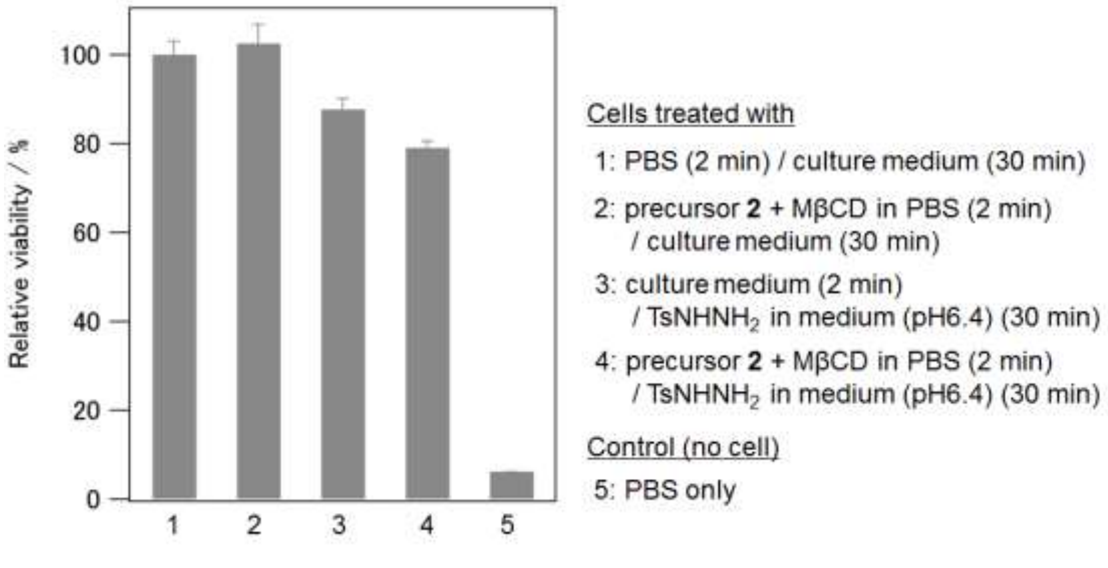

Figure S8. Relative viabilites of the cells treated with the chemically-activatable alkyne analogue and $TsNHNH_2$ compared to that of the cells treated with PBS and culture medium.

References

[S1] T. Hamada, Y Miura, K. Ishii, S. Araki, K. Yoshikawa, M. Vestergaard, and M. Takagi, *J. Phys. Chem. Lett. B*, 2007, **111**, 10853.

[S2] B. Lohse, P. Bolinger and D. Stamou, *J. Am. Chem. Soc.*, 2008, **130**, 14372.

[S3] Y. Ozeki, W. Umemura, Y. Otsuka, S. Satoh, H. Hashimoto, K. Sumimura, N. Nishizawa, K. Fukui and K. Itoh, *Nature Photonics*, 2012, **6**, 845.

[S4] A. Moscho, O. O. Daniel, T. Chiu, B. P. Modi and R. S. Zare, *Proc. Natl. Acad. Sci. U.S.A.*, 1996, **93**, 11443.
